# Post-transplant anemia as a persistent risk factor for graft dysfunction in kidney transplant recipients

**DOI:** 10.1080/0886022X.2026.2680735

**Published:** 2026-06-02

**Authors:** Makoto Tsujita, Yutaka Matsuoka, Kazuharu Uchida, Kunio Morozumi

**Affiliations:** ^a^ Department of Nephrology, Tajimi Clinic, Gifu, Japan; ^b^ Department of Kidney Disease Center, Masuko Kinen Byoin, Nagoya, Japan

**Keywords:** Post-transplant anemia, kidney transplantation, graft function

## Abstract

**Background:**

Post-transplant anemia (PTA) is associated with adverse graft outcomes after kidney transplantation. However, it remains unclear whether persistent anemia despite erythropoiesis-stimulating agent (ESA) therapy is associated with long-term graft dysfunction in routine clinical practice.

**Methods:**

This single-center retrospective study included 323 kidney transplant recipients followed at Masuko Kinen Hospital, Japan, from 2019 to 2021. The primary analysis used a linear mixed-effects model with repeated averaged estimated glomerular filtration rate (eGFRave) measurements over 2 years, treating hemoglobin (Hb) as a time-updated covariate. Supportive analyses evaluated 2-year change in eGFRave in the overall cohort and a propensity score-matched cohort.

**Results:**

Recipients receiving ESA therapy had lower Hb levels and worse baseline kidney function than those not receiving ESA therapy. In the primary linear mixed-effects model, eGFRave declined over time (*β* = −1.86 per year; 95% confidence interval [CI], −2.20 to −1.53; *p* < 0.001). Higher Hb levels were independently associated with higher eGFRave over time (*β* =  0.76 per 1 g/dL; 95% CI, 0.44–1.09; *p* < 0.001), whereas ESA use itself was not independently associated with graft function trajectory (*β* =  0.17; 95% CI, −0.80 to 1.14; *p* = 0.731). In supportive analyses, greater graft function decline was observed in recipients receiving ESA therapy in both the overall and propensity score-matched cohorts.

**Conclusion:**

Persistent PTA, rather than ESA use itself, was associated with graft function decline in kidney transplant recipients. Hb likely reflects underlying graft pathology, inflammation, or ESA resistance rather than being directly causal.

## Key messages

1. What is known
  Post-transplant anemia is common after kidney transplantation and has been associated with adverse graft outcomes. Although previous studies suggested that higher hemoglobin targets may be beneficial, the clinical significance of persistent anemia in routine practice remains unclear.
2. What this study adds
  In this single-center retrospective cohort of 323 kidney transplant recipients, higher time-updated hemoglobin levels were associated with better longitudinal graft function over 2 years, whereas ESA use itself was not independently associated with graft function trajectory. Additional analyses also supported the association between lower hemoglobin levels and greater graft function decline.
3. Potential impact
  Persistent post-transplant anemia, including persistent anemia despite ESA therapy, may help identify kidney transplant recipients at higher risk of subsequent graft dysfunction and may support closer follow-up and evaluation of potentially modifiable contributors to anemia in routine practice.

## Graphical Abstract

**Figure:**
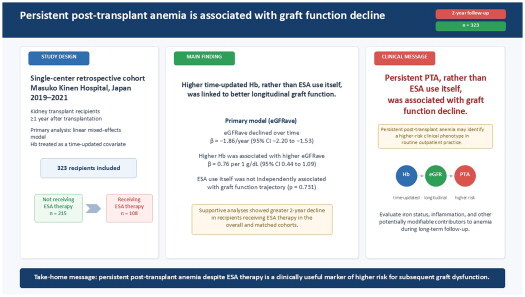

## Introduction

Even after successful kidney transplantation (KTx), post-transplant anemia (PTA) remains a common complication. The etiology of PTA is multifactorial, including graft dysfunction, rejection, iron deficiency, immunosuppressive therapy, and infections. PTA is associated with reduced exercise tolerance, cognitive decline, chronic fatigue, and decreased quality of life [1,2]. Furthermore, accumulating evidence suggests that PTA is negatively associated with long-term clinical outcomes in KTx recipients, such as graft failure, all-cause mortality, and progressive deterioration of graft function [2].

PTA is typically classified into early and late periods [3]. With regard to its impact on graft function, no randomized controlled trials (RCTs) have shown the optimal target hemoglobin (Hb) levels for better graft function in the early post-transplant period [4,5]. In the late post-transplant period, in a retrospective cohort study, Heinze et al. demonstrated that increasing Hb levels to above 12.5 g/dL with erythropoiesis-stimulating agents (ESAs) in KTx recipients was associated with increased mortality, and that this effect was significant at Hb concentrations above 14.0 g/dL [6]. However, our 3-year RCT demonstrated that higher Hb levels (Hb < 13.5 and ≥ 12.5 g/dL; mean achieved Hb: 12.8 g/dL) were associated with better graft outcomes compared to lower Hb levels (Hb < 11.5 and ≥ 10.5 g/dL). Mean difference in estimated glomerular filtration rate (eGFR) was −1.0 mL/min/m^2^ in high Hb group and −5.1 mL/min/m^2^ in low Hb group. Furthermore, there was no difference in cardiovascular events between the two groups, demonstrating the safety of the high Hb group [7]. Similarly, Choukroun et al. reported that patients in the normalization group (Hb 13–15 g/dL; mean achieved Hb: 13.4 g/dL) had more favorable outcomes than those in the partial correction group (Hb 10.5–11.5 g/dL) [8]. Both of these RCTs indicated that higher Hb levels were associated with improved graft survival.

The Japanese Guideline for Renal Anemia in Chronic Kidney Disease published in 2015 recommended a target Hb level of <13.0 g/dL in the late period. Based on these findings, Hb target levels (<13.0, ≥12.5 g/dL) were established to maintain graft function stability and have been applied in real clinical practice at Masuko Kinen Hospital since 2019. However, real-world data remain limited regarding whether persistent PTA despite ESA therapy is associated with subsequent graft dysfunction. Therefore, we examined the association between persistent PTA and graft function decline over 2 years in routine outpatient practice, including recipients receiving ESA therapy.

## Materials and methods

### Study design

A single-center retrospective observational study was conducted at Masuko Kinen Hospital in Japan to examine the association between persistent PTA and subsequent graft function trajectory in recipients ≥1 year after KTx. Patients were divided into those receiving and not receiving ESA therapy, and changes in kidney function over 2 years were compared between the two groups. Patients who needed ESA were given subcutaneous Epoetin Beta Pegol (Mircera^®^; Chugai Pharmaceutical Co. Ltd., Japan) 0–250 µg at each visit, and the doses and interval were adjusted according to Hb level and its target. (<13.0, ≥12.5 g/dL).

### Measurements of kidney function

Creatinine-based eGFR (eGFRcre) was calculated using the equation published by the Japanese Society of Nephrology [9]: 194 × (age)^−0.287^ × (sCr)^−1.094^, including a correction factor of 0.739 for women. Cystatin C-based eGFR (eGFRcys) was also calculated using the equation published by the Japanese Society of Nephrology [10]: (104 × cystatin-C^−1.019^ × 0.996 ^age^)-8 for men; (104 × 0.929 × cystatin-C^−1.019^ × 0.996 ^age^)-8 for women. Averaged eGFR (eGFRave) was calculated as the average of eGFRcre and eGFRcys. Our previous study demonstrated that eGFRave may better reflect estimated kidney function in Japanese recipients with KTx [10]. In this study, therefore, eGFRcre and eGFRave were assessed.

### Participants

**Figure 1.**
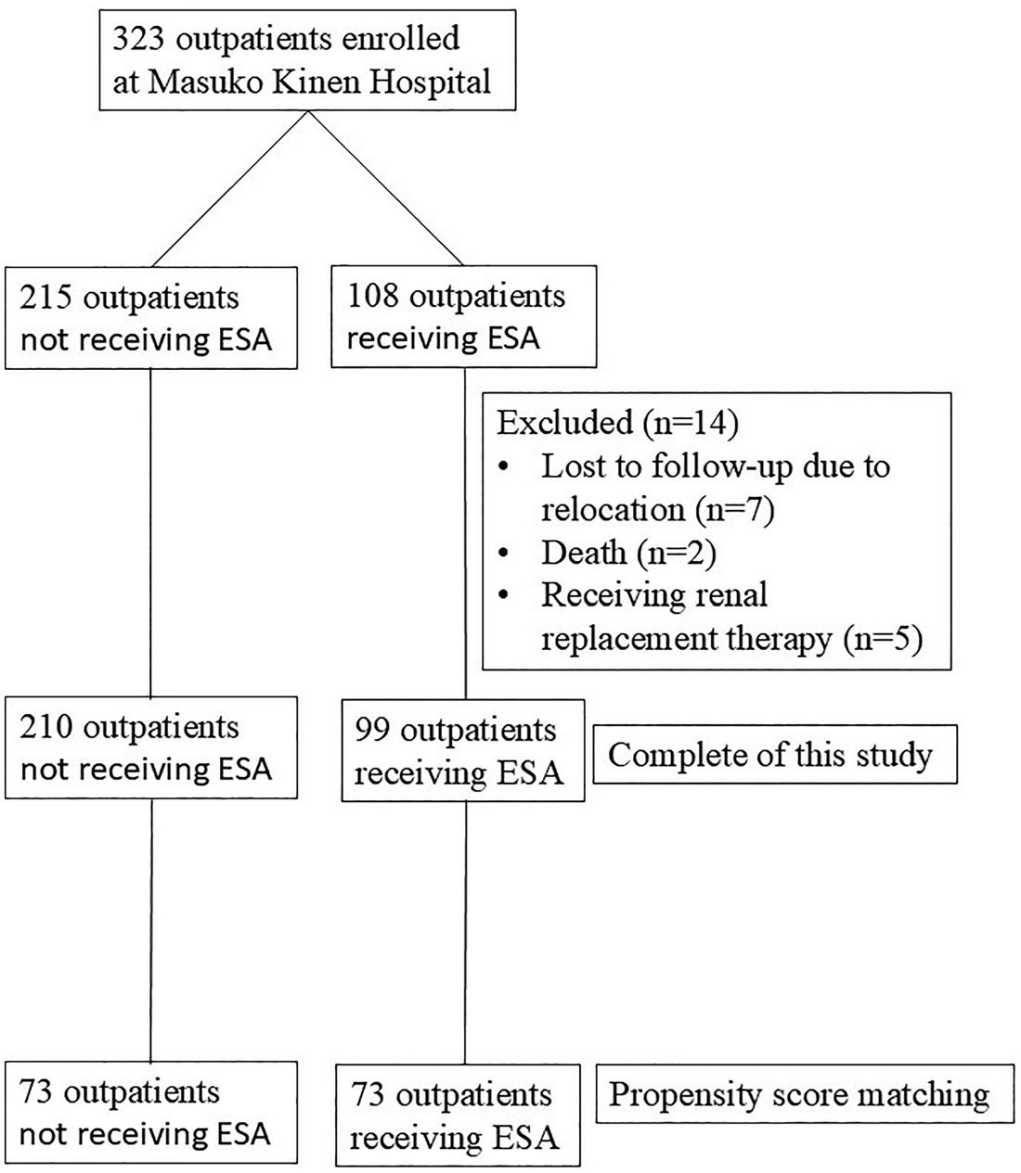
Flowchart of this study.

All outpatients at Masuko Kinen Hospital in 2019 were recruited. After excluding 14 patients, including 7 lost to follow-up due to relocation, 5 receiving renal replacement therapy (including 2 reinitiating dialysis), and 2 deaths, a total of 323 outpatients were enrolled in this study. Figure 1 shows the study flowchart.

The demographic and clinical characteristics of the patients were retrieved from the hospital electronic medical records. Informed consent for the data collection was waived due to the retrospective study design.

### Statistical analysis

Data were expressed as means ± standard deviation, 95% confidence intervals (CIs), or medians (interquartile ranges). Differences in variables between groups were evaluated using the Wilcoxon rank-sum test or paired or unpaired Student’s *t* test, as appropriate. The primary analysis used a linear mixed-effects model fitted to repeated eGFR measurements obtained at baseline and at 1 and 2 years. Time and Hb were included as fixed effects, with Hb treated as a time-varying covariate, and a patient-specific random intercept was included to account for within-patient correlation. Time was modeled as a continuous variable (0, 1, and 2 years), and a patient-specific random intercept was included to account for within-patient correlation. The model was adjusted for ESA use, age, sex, baseline eGFRave (time = 0), and baseline urine protein, which was included *a priori* as a fixed effect to account for baseline disease severity. As a supportive analysis, the same longitudinal model was also applied using eGFRcre.

Supportive analyses also included linear regression analyses to examine the association of baseline clinical factors with the 2-year change in kidney function. In addition, propensity score matching was performed to reduce differences in clinical and demographic characteristics between recipients receiving and not receiving ESA therapy. Propensity scores were estimated using a logistic regression model incorporating age, sex, eGFRave, transferrin saturation, and urinary protein. Matching was performed using 1:1 nearest-neighbor matching with a caliper width of 0.20, without replacement. The balance between groups before and after matching was assessed by descriptive comparisons of baseline characteristics. After matching, multivariable regression was performed as a supportive analysis to account for residual confounding and improve precision.

For a supportive subgroup analysis restricted to recipients receiving ESA therapy, persistent anemia was defined as Hb < 12.0 g/dL at two or more of the three assessments during the 2-year observation period. This cutoff was selected as a clinically pragmatic threshold for supportive subgroup analysis and was not intended as a definitive guideline-based threshold. In this subgroup, multivariable linear regression was used to examine the association between persistent anemia and the 2-year change in eGFRave after adjustment for age, sex, baseline eGFRave, and baseline Hb.

All *p* values were two-sided. A *p* value of <0.05 was considered statistically significant. All statistical analyses were performed using JMP version 17 (SAS Institute, Inc., Cary, NC).

## Results

### Baseline characteristics

The demographic and clinical characteristics of the study cohort are shown in Table 1. A total of 323 kidney transplant recipients were included, with 215 recipients not receiving ESA therapy and 108 recipients receiving ESA therapy. At baseline, recipients receiving ESA therapy had lower Hb levels (12.0 ± 0.9 *vs.* 13.4 ± 1.2 g/dL) and lower eGFRave (39.3 ± 13.6 *vs.* 51.1 ± 14.0 mL/min/1.73 m^2^) than those not receiving ESA therapy, indicating substantial baseline differences between the groups. After propensity score matching, most baseline characteristics became more comparable, although Hb levels remained lower in recipients receiving ESA therapy (Table 2). During the 2-year follow-up, no acute rejection episodes were identified in this cohort. Likewise, no cytomegalovirus or Epstein–Barr virus infection associated with anemia was identified during the study period.

**Table 1.** Characteristics of this study.

	Non-ESA (*n* = 215)	ESA (*n* = 108)
Male sex, *n* (%)	149 (69.3)	63 (58.3)
Age, years	51.2 ± 13.2	51.3 ± 13.4
Body mass index, kg/m^2^	23.3 ± 4.3	22.4 ± 5.3
Duration after KTx, months	120 [60, 192]	168 [116, 220]
Cause of chronic kidney disease, *n* (%) Diabetic nephropathy	8 (3.7)	4 (3.7)
Systolic blood pressure, mmHg	128.2 ± 8.7	127.6 ± 13.9
Laboratory examinations		
Hemoglobin, g/dL	13.4 ± 1.2	12.0 ± 0.9
Serum creatinine, mg/dL	1.30 ± 0.40	1.59 ± 0.61
Cystatin C, mg/L	1.49 ± 0.41	1.87 ± 0.65
eGFRcre, mL/min/1.73 m^2^	47.5 ± 12.6	38.3 ± 12.7
eGFRcys, mL/min/1.73 m^2^	55.0 ± 16.6	40.6 ± 15.9
eGFRave, mL/min/1.73 m^2^	51.1 ± 14.0	39.3 ± 13.6
Transferrin saturation, %	31.8 ± 11.6	40.2 ± 12.8
Bicarbonate, mmol/L	27.5 ± 3.0	26.1 ± 4.0
Intact parathyroid hormone, pg/mL	57 [41, 80]	63 [39, 95]
Corrected calcium, mg/dL	9.4 ± 0.4	9.4 ± 0.5
Phosphate, mg/dL	3.0 ± 0.5	3.3 ± 0.6
Urine protein, g/gCre	0.11 [0.07, 0.22]	0.18 [0.08, 0.38]
Immunosuppressive drugs		
Calcineurin inhibitor use, *n* (%)	215 (100.0)	108 (100.0)
Mycophenolate mofetil use, *n* (%)	207 (71.0)	85 (68.0)
Everolimus use, *n* (%)	52 (17.8)	22 (17.6)
Antihypertensive drugs		
Angiotensin II receptor blocker use, *n* (%)	169 (76.6)	92 (73.6)
Other antihypertensive drugs, *n* (%)	135 (46.2)	54 (43.2)
Statin use, *n* (%)	189 (64.7)	82 (65.6)
Dose of erythropoiesis stimulating agents (µg / month)	－	50 [25, 75]

Non-ESA: not receiving ESA therapy, ESA: receiving ESA therapy

**Table 2.** Characteristics of the study after propensity score matching.

	Non-ESA (*n* = 73)	ESA (*n* = 73)	*p* Value
Male sex, *n* (%)	46 (63.0)	46 (63.0)	1.000
Age, years	52.8 ± 13.5	50.5 ± 12.9	0.271
Body mass index, kg/m^2^	21.8 ± 3.1	22.5 ± 3.1	0.210
Duration after KTx, months	170 [107, 240.5]	180 [120, 220]	0.819
Cause of chronic kidney disease, *n* (%) Diabetic nephropathy	2 (2.7)	2 (2.7)	1.000
Systolic blood pressure, mmHg	130.0 ± 9.3	127.8 ± 10.2	0.180
Laboratory examinations			
Hemoglobin, g/dL	13.2 ± 0.9	12.0 ± 0.8	<0.001
Serum creatinine, mg/dL	1.47 ± 0.43	1.43 ± 0.41	0.561
Cystatin C, mg/L	1.63 ± 0.45	1.66 ± 0.46	0.692
eGFRave, mL/min/1.73 m^2^	43.7 ± 12.4	42.7 ± 11.5	0.614
Transferrin saturation, %	38.3 ± 11.6	37.2 ± 10.0	0.524
Bicarbonate, mmol/L	27.1 ± 3.4	26.4 ± 3.6	0.227
Intact parathyroid hormone, pg/mL	70 [39.5, 94]	62 [36, 87.5]	0.489
Corrected calcium, mg/dL	9.6 ± 0.5	9.4 ± 0.5	0.501
Phosphate, mg/dL	3.1 ± 0.6	3.2 ± 0.5	0.092
Urine protein, g/gCre	0.12 [0.08, 0.28]	0.19 [0.08, 0.37]	0.128
Immunosuppressive drugs			
Calcineurin inhibitor use, *n* (%)	73 (100.0)	73 (100.0)	1.000
Mycophenolate mofetil use, *n* (%)	49 (67.1)	51 (69.9)	0.729
Everolimus use, *n* (%)	15 (20.6)	15 (20.6)	1.000
Antihypertensive drugs			
Angiotensin II receptor blocker use, *n* (%)	50 (68.5)	54 (73.9)	0.465
Other antihypertensive drugs, *n* (%)	29 (39.7)	35 (48.0)	0.404
Statin use, *n* (%)	56 (76.7)	49 (67.1)	0.269

Non-ESA: not receiving ESA therapy, ESA: receiving ESA therapy

### Trends in Hb and eGFRave during this study in the overall cohort and in the propensity score-matched cohort

**Figure 2.**
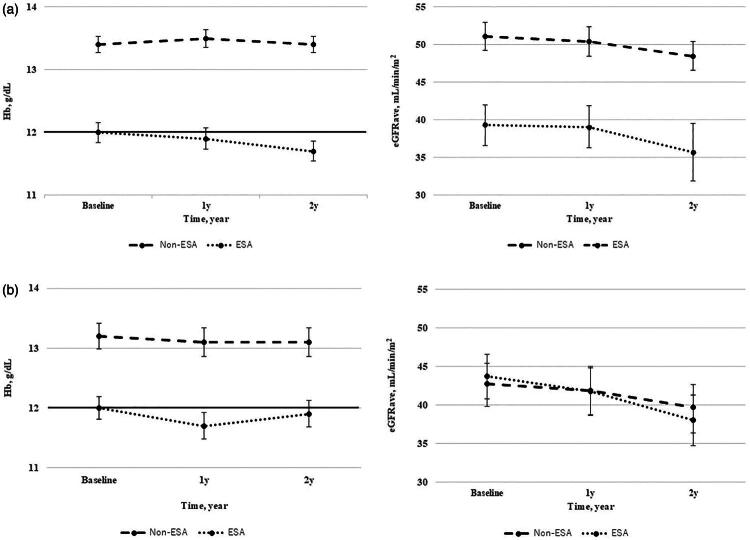
(a) Trends in Hemoglobin and eGFRave during this study pre-propensity score matching. The horizontal reference line in the hemoglobin panel indicates Hb 12 g/dL, which was used as a pragmatic threshold in the supportive analysis. Error bars represent 95% confidence intervals CIs. (b) Trends in Hemoglobin and eGFRave during this study post-propensity score matching. The horizontal reference line in the hemoglobin panel indicates Hb 12 g/dL, which was used as a pragmatic threshold in the supportive analysis. Error bars represent 95% confidence intervals (CIs). Non-ESA: not receiving ESA therapy, ESA: receiving ESA therapy.

In the overall cohort and the propensity score matched cohort, trends in Hb and eGFRave during this study are shown in Figure 2(a,b). In the overall cohort, the proportion of patients with a Hb level of ≥12 g/dL was 58.3% at baseline, 44.9% at 1 year, and 33.7% at 2 years.

### Primary longitudinal analysis using a linear mixed-effects model

In the linear mixed-effects model using eGFRave, eGFRave declined over time (*β* = −1.86 per year; 95% CI, −2.20 to −1.53; *p* < 0.001). Higher Hb levels were associated with higher eGFRave (*β* = 0.76 per 1 g/dL; 95% CI, 0.44–1.09; *p* < 0.001), whereas higher baseline urine protein levels were associated with lower eGFRave (*β* = −2.49; 95% CI, −3.60 to −1.38; *p* < 0.001). Baseline eGFRave was significantly associated with subsequent eGFRave measurements (*β* =  0.93; 95% CI, 0.90–0.96; *p* < 0.001). ESA use was not independently associated with eGFRave (*β* = 0.17; 95% CI, −0.80 to 1.14; *p* = 0.731). Similarly, age and sex were not independently associated with eGFRave (*p* = 0.811 and 0.335, respectively).

In the linear mixed-effects model using eGFRcre, similar findings were observed. eGFRcre declined over time (*β* = −1.56 per year; 95% CI, −1.90 to −1.23; *p* < 0.001), and higher Hb levels were independently associated with higher eGFRcre (*β* = 0.89 per 1 g/dL; 95% CI, 0.56–1.21; *p* < 0.001). Higher baseline urine protein levels were also associated with lower eGFRcre (*β* = −2.20; 95% CI, −3.30 to −1.10; *p* < 0.001), whereas ESA use was not independently associated with eGFRcre (*β*  = 0.38; 95% CI, −0.58 to 1.34; *p* = 0.439). Age and sex were likewise not independently associated with eGFRcre (*p* = 0.653 and 0.972, respectively).

### Changes in kidney function during 2 years in the overall cohort and in the propensity score-matched cohort

**Figure 3.**
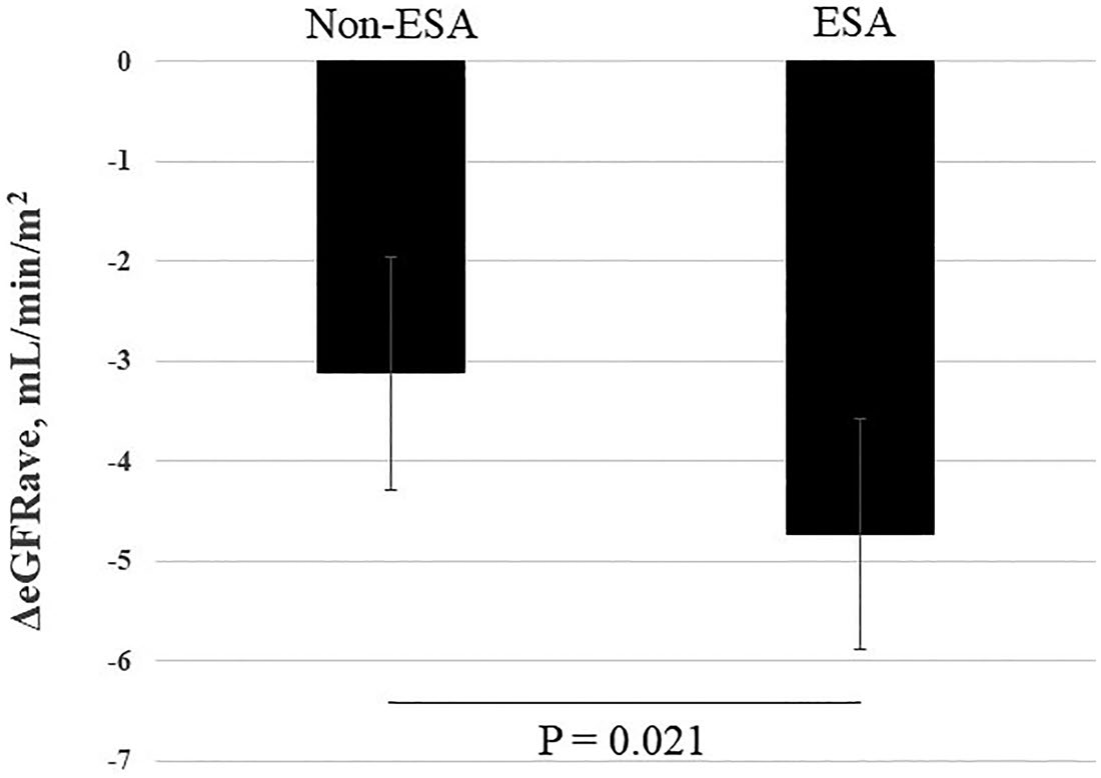
Changes in eGFRave during 2 years. Non-ESA: not receiving ESA therapy, ESA: receiving ESA therapy.

**Figure 4.**
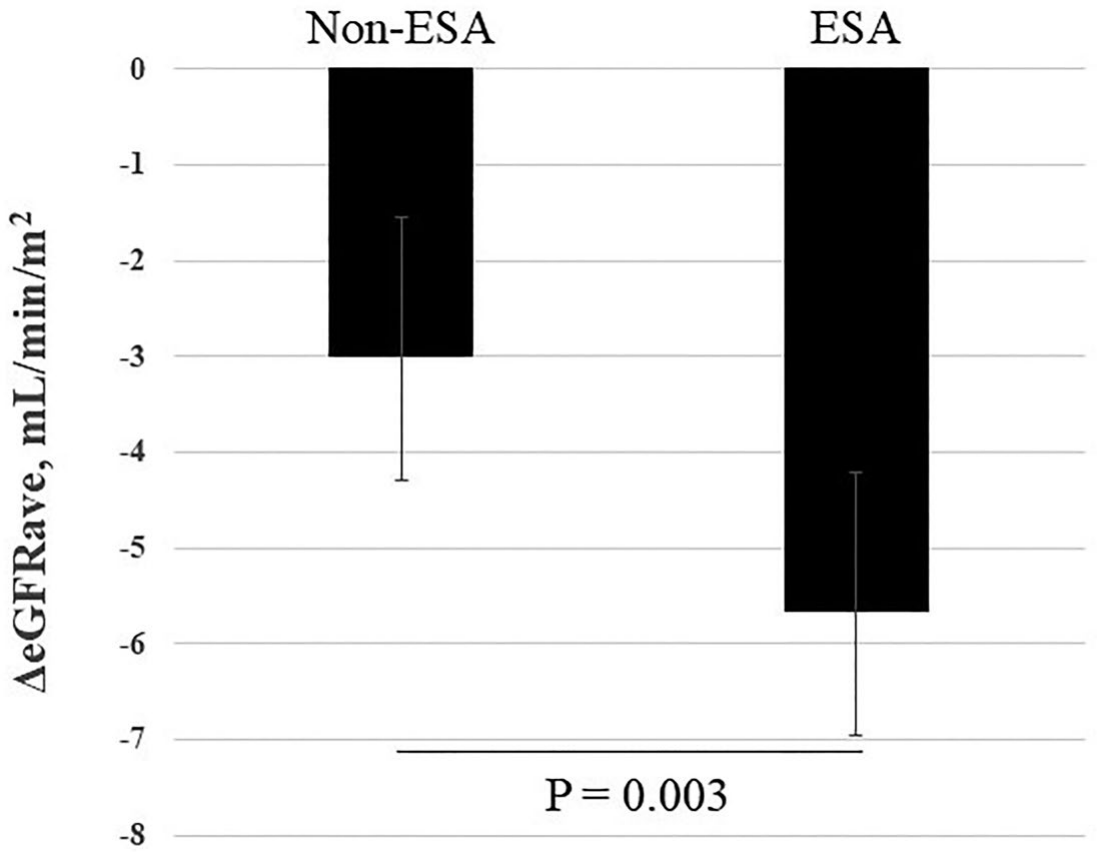
Changes in eGFRave during 2 years post-propensity score matching. Non-ESA: not receiving ESA therapy, ESA: receiving ESA therapy.

In the overall cohort, changes in eGFRave (ΔeGFRave, mL/min/1.73m^2^) during the 2 years of follow-up were −3.12 (95% CI, −3.89 to −2.36) in patients not receiving ESA therapy treatment and −4.73 (95% CI, −5.89 to −3.57) in those receiving ESA therapy, with a statistically significant difference (*p* = 0.021) (Figure 3). By multivariate analysis, Hb was a significant factor influencing ΔeGFRave, whereas ESA use did not have a significant impact on ΔeGFRave, as shown in Table 3. In the propensity score matched cohort (*n* = 146), ΔeGFRave were − 3.0 (95% CI, −4.1 to −1.93) and −5.66 (95% CI, −7.1 to −4.3) (*p* = 0.003, with adjustment for baseline eGFRave), as shown in Figure 4.

**Table 3.** Associations of clinical parameters at baseline with ΔeGFRave.

	Univariate			Multivariate		
	*β*	*t*	*p*	*β*	*t*	*p*
Age	0.03	0.49	0.628	0.01	0.17	0.866
Sex, female	0.11	2.01	0.045	0.09	1.66	0.098
Body mass index	0.09	1.51	0.131	0.11	1.84	0.066
Duration after KTx	−0.08	−1.39	0.165	−0.05	−0.85	0.395
Original Disease, DKD	−0.05	−0.23	0.504	−0.01	−0.23	0.821
Systolic blood pressure	−0.03	−0.51	0.613	−0.05	−0.83	0.409
Hemoglobin, baseline	0.17	3.02	0.003	0.16	2.28	0.023
eGFRave, baseline	− 0.08	−1.31	0.19	−0.27	−3.99	<0.001
Bicarbonate	0.06	1.07	0.287	0.12	1.9	0.059
PTH	−0.004	−0.08	0.938	0.02	0.41	0.683
Transferrin saturation	0.03	0.58	0.564	0.03	0.43	0.666
ESA use	−0.13	−2.33	0.021	−0.07	−0.96	0.338
Urinary protein	−0.25	−4.59	<0.001	−0.29	−5.13	<0.001

In a supportive analysis restricted to recipients receiving ESA therapy, persistent anemia, defined as Hb <12.0 g/dL at two or more of the three assessments during the 2-year observation period, was independently associated with a greater decline in eGFRave over 2 years (*β* = −3.26; 95% CI, −6.13 to −0.38; *p* = 0.027).

## Discussion

In the present real-world outpatient cohort of kidney transplant recipients ≥1 year after transplantation, the primary analysis using a linear mixed-effects model showed that higher time-updated Hb levels were independently associated with better longitudinal graft function, whereas ESA use itself was not independently associated with graft function trajectory. This association was observed after adjustment for time, baseline eGFR, age, sex, and baseline urine protein. In supportive analyses based on 2-year change in eGFR, recipients with lower Hb levels showed greater graft function decline in both the overall cohort and the propensity score-matched cohort. Furthermore, in a supportive analysis restricted to recipients receiving ESA therapy, persistent anemia was independently associated with a greater decline in eGFRave over 2 years. Taken together, these findings suggest that the clinically relevant phenotype is not ESA exposure itself, but persistent PTA, including persistent anemia despite ESA therapy, which may help identify recipients at higher risk of subsequent graft dysfunction in routine clinical practice. In this context, Hb likely reflects underlying graft pathology, inflammation, or ESA resistance rather than being directly causal.

Several RCTs have evaluated Hb targets in KTx recipients receiving ESA therapy, revealing that higher Hb targets are associated with improved renal functional outcomes in some settings [7,8,11], although optimal targets remain controversial. Based on this evidence, our center adopted a relatively higher Hb target in routine practice. However, in real-world outpatient care, achieving and maintaining such higher Hb levels proved challenging, and, on average, Hb levels remained lower in recipients receiving ESA therapy than in those not receiving ESA therapy. Therefore, this study cohort was not designed to evaluate the safety or efficacy of achieving a higher Hb target (e.g. ≥13 g/dL) through ESA therapy. Rather, our findings should be interpreted as associative, highlighting that persistent PTA despite ESA therapy may indicate a high-risk clinical phenotype associated with graft dysfunction.

The association between ESA use and graft function should be interpreted cautiously in our observational setting. ESA therapy is initiated and continued based on anemia severity; therefore, indication bias and residual confounding may have persisted despite propensity score matching and multivariable adjustment. Unmeasured factors, such as inflammation, iron-restricted erythropoiesis, ESA hyporesponsiveness/bone marrow resistance, and underlying allograft pathology, may contribute to both persistent anemia and graft dysfunction [2]. Importantly, Hb levels remained lower in recipients receiving ESA therapy even after propensity score matching, indicating that the matched comparison showed a difference between non-anemic recipients and those with persistent anemia despite ESA therapy. Accordingly, our findings do not establish a direct, causal detrimental effect of ESA but instead support an association, underscoring the clinical importance of identifying and characterizing persistent PTA in routine outpatient follow-up.

Prior trials evaluated prespecified Hb targets under controlled protocols [7,8,11], whereas our cohort reflects routine outpatient practice in long-term KTx recipients. Thus, our results complement the trial evidence by highlighting the prognostic relevance of persistent anemia in real-world care. Although eGFRave is not widely used internationally, we selected it *a priori* based on our previous validation study in Japanese kidney transplant recipients, in which it better reflected kidney function than creatinine-based eGFR alone. Nevertheless, generalizability to non-Japanese populations and other clinical settings remains uncertain.

From a practical standpoint, our findings indicate that persistent PTA, which often requires ESA therapy, may help identify recipients at higher risk of subsequent graft function decline during long-term outpatient follow-up. These results support closer monitoring of graft function and systematic evaluation of potentially modifiable contributors to anemia, such as iron status and the inflammatory milieu, while acknowledging that specific treatment strategies or Hb targets cannot be determined from this observational study. Accordingly, these findings should be interpreted as hypothesis-generating rather than practice-changing, because clinically important hard renal outcomes were infrequent during the 2-year observation period.

The observed association between lower Hb levels and graft dysfunction may reflect several overlapping pathways. Persistent anemia may reflect renal oxygen delivery and tissue hypoxia and can be accompanied by hemodynamic alterations; it may also serve as a surrogate marker of underlying processes that impair both erythropoiesis and graft function, such as chronic inflammation, iron-restricted erythropoiesis, and chronic allograft injury [2,12–14]. Because histopathology and comprehensive time-updated inflammatory assessments were not systematically available, the role of these mechanisms in our findings remains speculative, warranting further investigation. Because Hb may also lie on the pathway linking declining graft function, inflammation, and ESA treatment, the possibility of time-dependent confounding and reverse causation should be acknowledged. In particular, worsening graft function may itself contribute to subsequent anemia. Although the present longitudinal model was designed to better account for time-varying Hb levels, future studies using lagged analyses or marginal structural models may help further clarify the temporal and causal structure of this association.

This study has several limitations. First, this was a single-center retrospective study, which may limit the generalizability of our findings. Second, the sample size was modest. Third, Hb targets were not fully standardized and might have differed among the attending physicians. Fourth, residual confounding cannot be excluded, particularly because clinically important time-updated variables, such as ferritin, inflammatory markers, and histopathological findings, were not systematically available. Fifth, clinically important hard renal outcomes, such as graft failure or marked eGFR decline, were infrequent during the 2-year observation period, limiting event-based analyses. Sixth, the supportive analyses, including the propensity score-matched and ESA-restricted analyses, should be interpreted cautiously as complementary evidence to the main longitudinal findings. Finally, ESA exposure was modeled primarily as treatment status rather than as a quantitative variable; therefore, cumulative dose, dose intensity, and responsiveness to ESA were not fully evaluated.

## Conclusion

Persistent PTA, rather than ESA use itself, was associated with graft function decline in kidney transplant recipients. These findings suggest that Hb likely reflects underlying graft pathology, inflammation, or ESA resistance rather than being directly causal.

## Data Availability

The data supporting the findings of this study are available from the corresponding author upon reasonable request. The data are not publicly available due to privacy and ethical restrictions, in accordance with the Taylor & Francis Share upon Reasonable Request data policy.
